# mGem: Bacterial aversion to making new genes

**DOI:** 10.1128/mbio.02016-26

**Published:** 2026-10-09

**Authors:** Md. Hassan Uz-Zaman, Howard Ochman

**Affiliations:** 1 Department of Molecular Biosciences, University of Texas at Austin 196204, Austin, Texas, USA; Georgia Institute of Technology, Atlanta, Georgia, USA

**Keywords:** genome organization, gene repertoires, molecular evolution, *Escherichia coli*

## Abstract

All genes must ultimately have arisen *de novo* from non-coding sequences, but to what extent is this process an ongoing contributor to the contents of contemporary genomes? Based on the expansive pangenome of *Escherichia coli*, genes created *de novo* are short-lived and have made virtually no contribution to the set of longer, conserved genes that form the bulk of the canonical gene repertoire. Due to the generality of bacterial genome dynamics, this trend likely applies across taxa, suggesting that the vast majority of truly novel genes were created in the deep evolutionary past, thereby consigning the role of the newly invented genes to transient adaptive challenges.

## PERSPECTIVE

Bacterial species, even the strains within a bacterial species, can display very different gene inventories (1, 2). Bacterial genomes are tightly packed with genes, and there is an almost uniform association between genome size and gene number across species—from the 0.1-Mb genome of *Nasuia deltocephalinicola* to the 16-Mb genome of *Minicystis rosea*—with an average size of about one gene per kilobase (3).

The majority of genes in a genome are inherited directly from a parental cell, but beyond this strict vertical inheritance, there are other routes by which genes are furnished to bacterial genomes. Opportunities to expand gene content emerge from three processes: gene duplication, horizontal gene transfer, and *de novo* emergence from non-coding DNA (4). Whereas horizontal acquisition and, to a lesser extent, gene duplication are regarded as the primary contributors of new genes to bacterial genomes, the *de novo* emergence of genes from the non-coding portion of the genome—intergenic regions and alternative frames of coding regions—is considered a relatively rare process (5–8). However, every gene, if traced back far enough, must have originated *de novo* at some time in the past. To what extent is new gene formation an ongoing process in bacterial genomes?

## BACTERIAL GENES VS*.* GENE PRECURSORS

Access to full-genome sequences has fostered two approaches for evaluating the scope of *de novo* gene formation and its contribution to genome evolution (9). The first is to assess the sheer numbers and opportunities for new gene creation in terms of the potential ORFs, unanticipated transcripts, and novel proteins originating from a genome (10–17). The second tact considers taxonomically restricted genes confined to individual strains or species (a.k.a. “ORFans”) because the sporadic occurrence of such sequences can provide clues about how new genes originate (7, 8, 18–22).

Based on the translation start sites exposed by ribosome profiling, dozens (15, 17), if not thousands (14, 23, 24), of proteins could stem continually from non-genic regions of the genome (12). That unrecognized proteins could serve as the precursors of new functional genes is bolstered by large-scale functional screens of random peptides in which thousands of sequences of only 50 amino acids in length are capable of conferring a phenotype (25–28). Furthermore, studies in eukaryotes suggest intergenic ORFs—the non-coding substrates of *de novo* gene birth—are more likely than random sequences to encode functional proteins (29, 30). Therefore, not only are sizeable numbers of anonymous sequences being expressed and translated, but a significant fraction possesses latent functions—together emphasizing the plausibility of *de novo* gene emergence from previously non-coding sequences (31).

These findings point to an ongoing production of functional gene precursors often termed “proto-genes” (32), but the extent and manner by which proto-genes mature into *bona fide* genes are obscure. By and large, the broadly conserved genes that dominate bacterial genomes do not resemble proto-genes and are typically over five times longer than these putative gene precursors (17), a pattern that also extends to ORFans and lineage-specific genes, but to a lesser extent (8). This size disparity can be interpreted in several ways, which, in turn, concede different conclusions about the contribution of *de novo* gene birth to new gene evolution.

## DESTINY OF *DE NOVO* GENES

**Fig 1:**
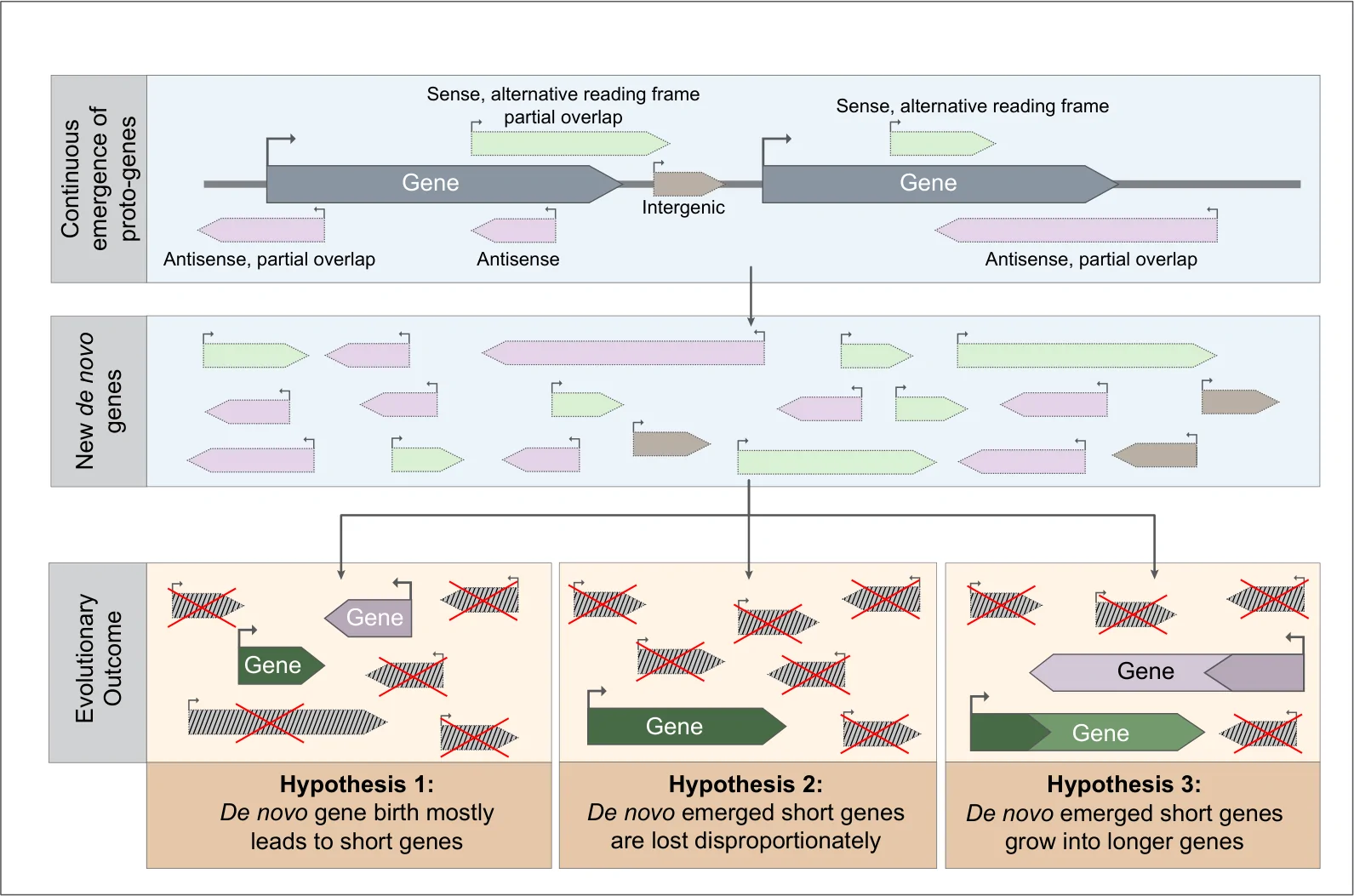
Emergence and fate of *de novo* genes. Dashed and solid lines indicate incipient *de novo* and conserved canonical genes, respectively. Each hypothesis presented in the bottom panels represents one of the three scenarios for the apparent disparity between conserved and *de novo* genes.

The first hypothesis contends that the size disparity between proto-genes and ancestral genes is an artifact because small genes have historically eluded recognition or functional characterization. Because bacterial protein-coding genes average a kilobase, gene annotations often disregard proteins shorter than a prescribed cutoff of 50 or 100 amino acids, which could lead to overlooking swathes of short, functionally relevant proteins (33). There has been a surge in the identification and characterization of small proteins, even in ostensibly well-annotated genomes, and many have been shown to be involved in membrane-related activities (34–39). For example, Fesenko et al. (23) identified 67,000 microprotein families bearing signatures of functionality in the genomes of enteric bacteria. As such, one possibility is that lineage-specific genes arise from the reservoir of short proto-genes and make up the lower tail of the size distribution of older conserved genes. In this scenario, the *de novo* gene birth contributes only to the pool of short genes due to their high rate of attrition (40–43), such that the vast majority of proto-genes are transient, and only a small fraction from the most prevalent size-class remains (Fig. 1).

A second hypothesis also assumes that gene loss is responsible for the size disparity between conserved and proto-genes, with the qualification that the shorter proto-gene sequences are disproportionately affected by this process. In this scenario, only *de novo* genes that form relatively long ORFs persist and become actual genes, a view that has been dubbed the “born this way” hypothesis (44, 45) (Fig. 1).

The third hypothesis posits that, over the course of time, new genes increase in length through fusion events, frameshifts, and/or remodeling initiation or termination signals—and eventually come to resemble the bulk of the conserved genes (Fig. 1). Many studies in eukaryotic systems suggest that older *de novo* genes (i.e*.*, those that emerged earlier and were more broadly distributed) tend to be longer, less disordered, and display a more compact structure than their younger counterparts (46–48). In this scheme, *de novo* genes proceed toward the properties of conserved genes as they mature; however, it could also be viewed as providing some support to hypothesis 2, such that the genes that lack these features are disproportionately eliminated through time.

In light of the disparate features of young and old genes, we reasoned that analysis of the contents of contemporary genomes might help distinguish among these hypotheses. We focus on *Escherichia coli* because the species encompasses one of the largest pangenomes, which greatly increases the prospect of incorporating strain- and lineage-specific *de novo* genes. We sampled the available genome down to a large number of strains that together represent the diversity of the species at large, and focusing on these genomes enabled us to leverage our previous findings about the frequency of new gene birth in bacteria (8; see File S1).

## NO CONCLUSIVE SUPPORT FOR ANY OF THE THREE HYPOTHESES

The difference between the high number of gene precursors and the rarity of new genes in bacterial genomes attests to their high rate of loss (17, 24). Across the entire 31,277-gene-family pangenome of *E. coli*, we detected fewer than 20 cases of species-specific genes that could be traced to a *de novo* origin from resident sequences (8). In contrast, the bulk of the 260 ORFan-gene-families originated by horizontal transfer from distant sources (File S1). Almost all *de novo* genes are confined to a single strain or genome (but in a single case, one was present in two strains), and while four exceeded 100 amino acids, none approached the length of a typical bacterial gene.

The scarcity of *de novo* genes points to a high rate of proto-gene loss and may look to favor hypotheses 1 and 2; however, the extremely narrow distributions of every *de novo* gene signal that they are not maintained and do not become integrated into the gene repertoire of the species at large. Based on our knowledge of bacterial genome dynamics, such an outcome is expected: bacterial genomes suffer from a high rate of deletional bias that quickly removes superfluous gene precursors or genes with minimal functionality (49, 50).

It is possible, as Hypothesis 3 prescribes, that the lack of older, more widely distributed *de novo* genes is due to their transformation into more typical genes. The postulated transition of short genes into longer genes can be tested directly by examining whether *de novo*-emerged genes have either acquired new domains or incorporated flanking non-coding regions via frameshifts and translation read-through. If such cases exist in contemporary bacterial genomes, remnants of short precursors will be detectable through homology searches, so we applied a strategy that identified all currently recognized proteins in the *E. coli* pangenome that are either themselves short or exhibit homology with short proteins.

Of the 6,478 protein families in the pangenome with lengths below 100 amino acids, only 77 were found to have given rise to longer proteins (>250 amino acids) in any other bacterial genome, and just 0.2% of long *E. coli* proteins (25 out of 11,601 families) were found to have been derived from a short precursor. Putting this into perspective, fewer than 0.4% of the genes across the entire *E. coli* pangenome were involved in a significant gene expansion process, whereas ~99.6% have remained of similar length, possibly since their origination millions to billions of years ago (File S1).

Such search strategies are naturally premised on the detectability of ancestral short homologs of contemporary genes, and such analyses might overlook some very ancient gene expansion events due to the decay of sequence homology (51). But, the fact that we find so few cases of recent expansions underscores the rarity of such events within the long timeframe captured by detectable evolutionary divergence.

Taken together, our analyses suggest that *de novo* gene birth has rarely, if ever, led to the birth of the canonical genes currently residing in *E. coli* genomes either by long-term persistence of newly formed long ORFs or by the expansion of shorter precursor genes.

## *DE NOVO* GENE BIRTH AND BACTERIAL INNOVATION

The process of *de novo* gene birth has not completely perished—bacterial genomes constantly generate proto-genes, which can act as prospective substrates for new genes. Bacterial genomes often encode short proteins that are involved in membrane, defense, or regulatory roles (35, 37), and some fraction of these genes might have *de novo* origins. Furthermore, analyses relying on gene annotations alone may miss short and lineage-specific genes, and previous work on proto-genes (17) or intergenic microproteins with functional signatures (24, 25) have suggested a significant proportion of these unannotated sequences emerged *de novo*.

Most often, however, bacteria rely on lateral gene transfer to expand their genomic inventories without having to create entirely new genes. The recesses filled by *de novo* genes appear to involve a rapid and short-term response to a new ecological challenge, such as the production of toxins involved in intra- or interspecific competition or in the fine-tuning of a regulatory response, roles often best served by short proteins with minimal structural complexity.

Beyond these transient genetic innovations, we find no evidence that the bulk of *de novo* genes are maintained in the long term, or that canonical bacterial genes—those that are longer, form stable, tertiary-folded structures, and often perform catalytic reactions or become incorporated into metabolic pathways—have emerged *de novo*. When considered together with the understanding that all genes must have ultimately emerged *de novo*, our findings indicate that such events must be relegated to the very distant past. Over a decade ago, David and Alm (52) described a burst of new gene birth that coincided with the Great Oxidation Event about 3 billion years ago and waned by an estimated billion years ago. Their phylogenetic approach was incapable of considering lineage-specific ORFan genes—the category that would include evidence of more recent bouts of *de novo* gene birth—but ours expose a subsequent lack of such events during the subsequent diversification. *E. coli* has a large and complex pangenome and has been extensively sampled, features that make it a prime candidate for both the formation and the detection of new genes. However, considering the generality of the underlying processes governing gene gain and loss in bacteria, the striking lack of *de novo* genes in this expansive pangenome suggests that this feature is characteristic of all bacterial taxa.

## SUPPLEMENTAL MATERIAL

File S1 mbio.02016-26-s0001.docx Supplemental methods.

ASM does not own the copyrights to Supplemental Material that may be linked to, or accessed through, an article. The authors have granted ASM a non-exclusive, world-wide license to publish the Supplemental Material files. Please contact the corresponding author directly for reuse.
