# Supplementary material for: mGem: Bacterial aversion to making new genes
Source: mBio. 2026 Oct 9;17(11):e02016-26. doi: 10.1128/mbio.02016-26 (PMC13753837; doi:10.1128/mbio.02016-26)
Supplement: Supplementary file 1 [file mbio.02016-26-s0001.docx]

**Supplementary Methods**

The *E. coli* pangenome was constructed by first grouping all complete genomes labelled as *Escherichia coli* in RefSeq into 99.5% average nucleotide identity clusters. We retained the 51 largest clusters and selected ten representative genomes from each for subsequent analyses, which together comprise over 76% of the entire set of genomes. We then annotated the genomes and clustered their proteins into 30,552 families using **MMSeqs2** and **SiLiX** (percent identity 60%, alignment coverage 60%) (1, 2).

From the pangenome rendered from this set of 510 genomes, we sought the two possible routes of the gene-length expansion process: (1) short *E. coli* proteins (<100 amino acids) with a longer homolog in outgroup genera; and (2) long *E. coli* proteins (>250 amino acids) that have a shortened version outside of *Escherichia* (applying the higher length cutoff corresponding to the average protein length in *E. coli*). In the first scenario, the shorter protein in *E. coli* is considered the ancestral version, which has expanded in other taxa; and in the second scenario, the longer protein in *E. coli* denotes the expanded derived state. All proteins having at least one outgroup homolog of divergent length were considered. Homology searches were conducted with **Diamond** (3).

Because ancestral homologs of a gene tend to be broadly conserved, we exclude cases in which short proteins are genus-specific and limited to a minority of genomes in their respective genera. To remove cases of annotation errors, we further excluded proteins whose derived, longer variants only appeared in two or fewer genomes. All remaining sets of homologous proteins were subjected to phylogenetic reconstruction to infer the ancestral state. For this purpose, each genus was assigned a state depending on the size of the homolog in a majority of its genomes: short (< 100 amino acids), intermediate (100 – 250 amino acids) or long (> 250 amino acids). For each candidate, we pruned the bacterial GTDB tree to only include those genera in which the protein is present and inferred the ancestral state using Ancestral Character Estimation (**ace**, implemented in the **ape** package in R) (4). Cases in which a short ancestral homolog was inferred with a >50% probability were retained.

Detection of *E. coli*-specific ORFans and *de novo* genes was carried out following the methodology in (5).

**References**

1.	Steinegger M, Söding J. 2017. MMseqs2 enables sensitive protein sequence searching for the analysis of massive data sets. *Nat Biotechnol* 35:1026–1028.

2.	Miele V, Penel S, Duret L. 2011. Ultra-fast sequence clustering from similarity networks with SiLiX. *BMC Bioinformatics* 12:116.

3.	Buchfink B, Reuter K, Drost H-G. 2021. Sensitive protein alignments at tree-of-life scale using DIAMOND. *Nat Methods* 18:366–368.

4.	Paradis E, Schliep K. 2019. ape 5.0: an environment for modern phylogenetics and evolutionary analyses in R. *Bioinformatics* 35:526–528.

5.	Uz-Zaman MH, Ochman H. 2026. Imported, not invented, genes prevail among *Escherichia coli* ORFans. *Proc Natl Acad Sci U S A* 123:e2523357123.
